# The impact of cellular senescence on aging skeletal muscle

**DOI:** 10.3389/fcell.2025.1719279

**Published:** 2025-11-28

**Authors:** Michael Kamal, Ryan Bevington, Amanda Johnson, Gianni Parise

**Affiliations:** Exercise Metabolism Research Group, Department of Kinesiology, McMaster University, Hamilton, ON, Canada

**Keywords:** cellular senescence, aging, skeletal muscle, regeneration, satellite cells, SASP, senolytics

## Abstract

Skeletal muscle is a highly plastic tissue that relies on its resident muscle stem cell population, known as satellite cells (MuSC), for its timely repair and regeneration. During aging, there is a decline in muscle regenerative capacity that is largely attributed to the loss of MuSC content and function. These aberrations are thought to contribute to the aging-related decline in skeletal muscle mass and strength. Cellular senescence, which is characterized by a state of irreversible cell cycle arrest and the presence of a senescence-associated secretory phenotype (SASP), has emerged as a potential factor in the dysfunction of MuSCs with aging. Much effort has recently been made to examine the detrimental effects of senescence on skeletal muscle as well as identify therapeutic approaches to selectively eliminate these cells and improve the aging phenotype. Here, we discuss the current understanding of aging-related MuSC impairments and the underlying mechanisms that link cellular senescence to the decline in muscle regenerative capacity.

## 1 Introduction

As humans age, we experience declines in overall health and organ-specific function throughout our bodies. Certain factors, such as genomic instability, stem cell exhaustion, and mitochondrial dysfunction, contribute to these progressive and inevitable changes (López-Otín et al., 2023). Skeletal muscle, as a highly dynamic and metabolic tissue, is particularly susceptible to the progressive decline in molecular and cellular function that occurs with advanced age, which could eventually lead to sarcopenia and frailty. These diseases cause a significant loss of independence and are major risk factors for developing aging-associated comorbidities (Cruz-Jentoft and Sayer, 2019; Pacifico et al., 2020; Sasaki and Fukumoto, 2022). Several mechanisms have been previously identified that actively contribute to muscle dysfunction with aging, but recently, research into cellular senescence has revealed a potentially large role for this fundamental biological process (Granic et al., 2023; Muñoz-Espín and Serrano, 2014). Cellular senescence, the irreversible arrest of cell proliferation, is a physiological stress response and a known hallmark of aging. When cells are unable to adequately respond to damaging stimuli, internal homeostatic machinery can force the cell into a state of senescence or premature apoptosis (Collado et al., 2007; Van Deursen, 2014). Numerous studies over the last decade have linked senescence to aging-related muscle impairments, indicating that our understanding of how cellular aging drives these deficits is still limited and requires further investigation (Zhang X. et al., 2022; Englund et al., 2021; He et al., 2021). This review aims to discuss the unique biological processes involved with cellular senescence, and how these features can influence skeletal muscle dynamics during periods of damage and regeneration. Specifically, we will highlight the known mechanisms that contribute to the aging-related dysfunction of muscle stem cells, the essential hallmarks of senescence and the SASP, the current evidence implicating senescence in skeletal muscle aging, and the implications of clearing senescent cells from the muscle microenvironment.

## 2 Muscle stem cells and aging

Under healthy, homeostatic conditions, muscle stem cells or satellite cells (MuSCs) are quiescent until activated in response to muscle damage, after which they differentiate to repair and regenerate the injured tissue. The naturally quiescent status of MuSCs makes them especially susceptible to the accumulation of intracellular damage that occurs during aging, ultimately resulting in a decrease in both MuSC number and function (Sousa-Victor and Muñoz-Cánoves, 2016). These detriments have led to impairments in the regenerative capacity of aged muscle (Conboy and Rando, 2005). While the change in MuSC characteristics is well documented in aged humans and animals, there is still much debate about the extent to which the MuSC perturbations with aging contribute to sarcopenia. Previous work has shown that MuSC depletion in sedentary mice does not affect muscle size during aging despite lower regenerative capacity (Fry et al., 2015; Keefe et al., 2015). On the other hand, there is evidence that MuSC content is associated with the regulation of muscle mass during aging. One group found that the depletion of MuSCs limits the hypertrophic growth response to lifelong physical activity in aged mice (Englund et al., 2020). Furthermore, it is well known that MuSCs in extraocular muscles are maintained in aging and muscle dystrophy, which is accompanied by the sparing of these muscles from the associated muscle wasting (Formicola et al., 2014; Kaminski et al., 1992). More recently, published work implicated the fibrogenic conversion of MuSCs as a significant component of sarcopenia and demonstrated that melatonin treatment replenishes the MuSC pool and mitigates the loss of muscle mass and strength in aged mice (Zhu et al., 2024). Unfortunately, there is still no definitive consensus on whether satellite cells significantly contribute to sarcopenia in humans. There is inherently a relationship between MuSCs and sarcopenia, but whether the loss or dysfunction of these cells substantially contributes to the progressive decline of skeletal muscle remains to be seen.

### 2.1 Mechanisms of aging-related MuSC dysfunction

The decline in MuSC content in old muscle tends to occur primarily in type II-associated MuSCs, often correlating with the substantial atrophy of type II myofibres observed with advancing age (Verdijk et al., 2007; Verdijk et al., 2014). Several mechanisms have been proposed as contributing to this loss, primarily revolving around the concept of stem cell exhaustion (Sousa-Victor et al., 2021; Ancel et al., 2021). The aged MuSC niche contains elevated levels of fibroblast growth factor (FGF), which has the unfortunate effect of driving a subset of unstimulated MuSCs to leave their quiescent state and lose their self-renewing capacity (Chakkalakal et al., 2012; Pawl et al., 2017). This is particularly detrimental in aged muscle because any damaging stimulus would be accompanied by a failure to replenish the basal MuSC pool and result in lower MuSC content throughout the muscle. Moreover, research conducted in both rodents and humans has shown that aged MuSCs are more susceptible to apoptosis than their young counterparts (Fulle et al., 2013; Jejurikar et al., 2006). Taken together, these data imply that decreases in MuSC content with aging are linked to a loss of quiescence, diminished self-renewal, and elevated cell death.

While there is a discrepancy in the field regarding the implication that diminished MuSC content significantly contributes to sarcopenia, it is possible that the functional detriments of aged MuSCs may augment the aging-associated pathology of skeletal muscle. Several molecular and metabolic perturbations contribute to the decline of MuSC function in advanced age. When healthy cells accumulate damaged organelles and components, they activate processes like autophagy to remove these materials and restore homeostasis. Like many other organs and tissues, MuSCs experience impairments in autophagy with aging, which causes a significant decline in regenerative capacity due to this loss of proteostasis (García-Prat et al., 2016a). Diminished autophagic clearance of damaged cellular components leads to the accumulation of intracellular debris and damaged mitochondria, which ultimately transitions MuSCs from a state of quiescence (reversible cell cycle arrest) to one of premature senescence (irreversible cell cycle arrest) or apoptosis (García-Prat et al., 2016b; White et al., 2018). This would also explain the inability of aged MuSCs to maintain quiescence and the decline in MuSC content with aging that was previously discussed. In addition to the reduced mitochondrial autophagy (mitophagy), aged MuSC also experience an imbalance in mitochondrial remodelling, resulting in a loss of oxidative phosphorylation (OXPHOS); crucially, genetically restoring OXPHOS or mitophagy in these cells enhances muscle regeneration (Hong et al., 2022). Further discussion on the mechanisms of senescence induction can be found in Section 3.

Cell cycle kinetics are also disrupted in aged MuSCs. Impaired mitophagy causes an increase in intracellular reactive oxygen species (ROS) levels, which in turn upregulates the expression of p16^INK4a^, the cyclin-dependent kinase inhibitor (CDKI) (García-Prat et al., 2016b; Passos et al., 2007). Cyclins, a class of proteins involved in cell cycle progression, are implicated in normal myogenic proliferation and differentiation (Mademtzoglou and Relaix, 2022). Alterations in CyclinD1 levels have been observed in aged MuSCs, although there is very limited research on this topic (Brett et al., 2020; Kimmel et al., 2020). Moreover, the loci of p16 and p21 are more epigenetically silenced in young MuSCs compared to old (Li et al., 2015). This could explain why MuSCs in very old muscles experience a derepression of p16, which pushes them out of quiescence into irreversible cellular senescence (Sousa-Victor et al., 2014). The relationship between senescence and aging muscle satellite cells will be discussed further in the following sections. The molecular detriments described in this review are implicated in aging-associated MuSC dysfunction, but it remains unclear whether they are a direct consequence of the aging environment or if they independently develop over time and, in turn, exacerbate the aging phenotype.

### 2.2 Factors that influence satellite cell fate during aging

Genetic heterogeneity between MuSC populations often dictates cell fate during muscle regeneration and aging. As discussed above, the myogenic regulatory factors (MRFs) contribute to the progression of MuSC through the myogenic program. MuSCs that are Myf5^–^ are more stem-like and thus replenish the MuSC niche, whereas Myf5^+^ MuSCs are more committed to differentiation (Kuang et al., 2007). Cells lacking Myf5 or its MRF companion MyoD display very mild differentiation, while the genetic double knockout (dKO) of both MRFs completely impairs it (Yamamoto et al., 2018; Rudnicki et al., 1993; Megeney et al., 1996). Furthermore, aged mice with MyoD/Myf5^dKO^ MuSCs could not regenerate properly after injury (Yamamoto et al., 2018). Another study reported that MuSCs expressing high levels of Pax7 are more dormant, take longer to respond to injury and have lower metabolic activity than Pax7^Low^ MuSCs (Rocheteau et al., 2012). Cell surface markers can also represent different populations of MuSCs with distinct behaviours. CD34^High^ MuSCs are more stem-like and less likely to commit to myogenic differentiation than CD34^Low^ cells, not unlike the pattern observed with Pax7 expression (García-Prat et al., 2020). Surprisingly, the CD34^High^ quiescent preference is preserved during aging, with these MuSCs remaining dormant and only being activated at very advanced ages due to the elevation of pro-myogenic signalling (García-Prat et al., 2020). This is consistent with recent work that showed CD34^+^ MuSCs are persistently quiescent in culture and capable of maintaining their phenotype with very limited proliferation (Steele et al., 2024). These findings indicate that intrinsic factors, such as molecular heterogeneity, are directly implicated in the activity and stemness of MuSCs.

MuSCs can undergo transcriptomic and metabolic reprogramming based on their microenvironment (Sousa-Victor et al., 2021). Some of the earliest evidence of this utilized heterochronic parabiosis to show that exposing aged muscle to young, healthy serum improved MuSC function and restored muscle regenerative capacity (Co et al., 2005). MuSC within extraocular muscles (EOM) are molecularly distinct from their limb-derived counterparts and are preferentially spared from dysfunction during aging or muscular dystrophies (Formicola et al., 2014; Kaminski et al., 1992; Evano et al., 2020). Notably, transplanting EOM MuSCs into tibialis anterior (TA) muscles caused them to adopt a TA-like transcriptomic profile, indicating the molecular identity of these cells was primarily driven by the limb muscle MuSC niche (Evano et al., 2020). Even more drastically, previous work has demonstrated that the *in vitro* treatment with bone morphogenic proteins or adipogenic inducers can induce the osteogenic and adipogenic conversion of adult murine MuSCs (Asakura et al., 2001; Wada et al., 2002). These studies showcase the remarkable plasticity of these stem cells and suggest that the aging-related MuSC perturbations are more likely driven by extrinsic factors present in the muscle microenvironment rather than intrinsic defects in cellular components.

## 3 Cellular senescence–a primer

The first *in vitro* cell culture experiments occurred in the early 20th century when scientist Ross Harrison isolated and expanded nerve cells using explanted frog embryonic tissue (Har et al., 1907). The use of isolated cells, *in vitro*, expanded rapidly over the subsequent decades, and researchers around the world began developing techniques to isolate and maintain cells in culture and establish cell lines from animal and human tissues. One of the most significant discoveries came in 1961 when Leonard Hayflick and Paul Moorhead found that human fetal fibroblasts could undergo a finite number of population doublings before ultimately experiencing proliferative arrest (Hayflick and Moorhead, 1961). This finding challenged the current dogma that all cells were immortal and gave rise to the theory that normal cells had a finite replicative capacity, while cancer cell lines could proliferate indefinitely. Hayflick coined the term “cellular senescence” to describe the phenomenon of cell replicative arrest, and his seminal work began the study of aging mechanisms within cultured cells (Hayflick and Moorhead, 1961; Khokhlov, 2013). Today, senescence can be briefly defined as the irreversible arrest of the cell cycle in metabolically active and living cells.

### 3.1 Senescence and cellular aging

Aging is accompanied by a decline in health and function that impacts every organ throughout the body. Several cellular and molecular characteristics are often observed with advancing age that negatively impact tissue homeostasis, such as genomic instability, mitochondrial dysfunction, altered signalling, and stem cell exhaustion, to name a few (López-Otín et al., 2023). Many aging-related perturbations are brought about by the inability of cells to adequately respond to these stimuli, which usually forces them into either cellular senescence or apoptosis. Over time, this results in the accumulation of senescent cells across multiple organs and tissues, which can initiate or exacerbate age-related disorders (Van Deursen, 2014). While premature senescence induction can produce deleterious effects, the timely initiation of this mechanism does have a beneficial role in several biological functions, including embryonic development, wound healing, and tumour suppression (Collado et al., 2007; Demaria et al., 2014; Muñoz-Espín et al., 2013). The dual nature of cellular senescence is a prime example of the concept of antagonistic pleiotropy, which states that beneficial traits found in young organisms can become detrimental later in life (Wiley and Campisi, 2021).

The phenomenon discovered by Hayflick and Moorhead can now be more accurately termed “replicative senescence.” The underlying cellular mechanism behind this discovery was largely attributed to the gradual erosion of telomeres that occurred with each division (Bodnar et al., 1998). Telomeres, which are short repeat nucleotide sequences found at the end of chromosomes, protect against chromosomal deterioration during transcription. After every round of replication, DNA polymerases leave a few bases at the end of the telomere unreplicated, and for most cells, this results in the erosion of the telomeres with each cell cycle (Campisi, 1997). Fortunately, some cells express telomerase, a protective enzyme that adds telomeric repeats to chromosome ends and prevents DNA damage (Campisi, 1997). However, if telomeres reach a critical length, they trigger a DNA damage response (DDR) that induces replicative senescence (d’Adda di Fagagna et al., 2003).

For many years, replicative senescence was the sole focus within the field of cellular aging. Eventually, researchers began to observe that several other cellular perturbations are capable of inducing senescence independent of telomere erosion, such as mitochondrial dysfunction, epigenetic modifications, non-telomeric DNA damage, loss of proteostasis, oncogene activation, chronic inflammation, and oxidative stress (Chandeck and Mooi, 2010; Höhn et al., 2017; Miwa et al., 2022; Sidler et al., 2017; Wen and Klionsky, 2016). Even adjacent senescent cells were observed to cause paracrine senescence in nearby healthy cells by releasing pro-inflammatory factors as part of the senescence-associated secretory phenotype (SASP) (Acosta et al., 2013). For the purposes of this review, we will refer to the premature (*i.e.,* non-replicative) senescent state as “stress-induced senescence”. Notably, several of these stressors are also considered hallmarks of aging (López-Otín et al., 2023), and the clearance of senescent cells in aged mice can extend both healthspan and lifespan (Xu et al., 2018). Taken together, this positions cellular senescence as a foundational component of age-related dyshomeostasis that is not just a byproduct of aging, but causative as well.

### 3.2 The intracellular senescence phenotype

**FIGURE 1:**
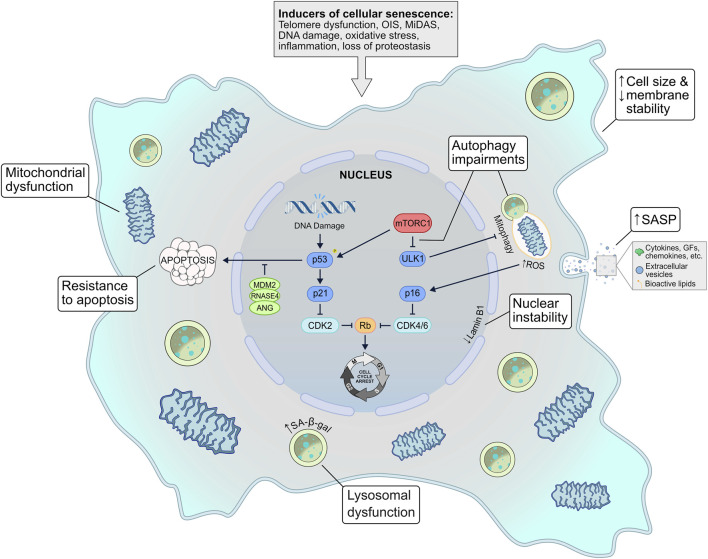
The inherent mechanisms of cellular senescence. Several possible stimuli can induce a senescent phenotype–telomere dysfunction, oncogene signalling, mitochondrial dysfunction, genotoxic or oxidative stress, inflammation, and loss of proteostasis. In response to these triggers, several signalling pathways may be activated, such as the p53-p21 and the p16^INK4a^-Rb axes, which ultimately invoke cell cycle arrest. Senescent cells exhibit mitochondrial dysfunction that is often exacerbated by autophagic (i.e., mitophagic) disruption and elevated ROS levels. Other common characteristics include lysosomal dysfunction, membrane/nuclear instability, and resistance to apoptosis. A defining feature of senescent cells is the senescence-associated secretory phenotype (SASP), a diverse secretome of cytokines, chemokines, growth factors, extracellular vesicles, and bioactive lipids that can act in an autocrine and paracrine fashion to reinforce senescence signalling. EV, extracellular vesicle; GF, growth factors; MiDAS, mitochondrial dysfunction–associated senescence; OIS, oncogene-induced senescence; ROS–reactive oxygen species; SA-β-gal, senescence-associated β-galactosidase; SASP, senescence-associated secretory phenotype.

Our understanding of cellular senescence has advanced greatly since the term was first introduced over 60 years ago. It is now apparent that the senescent phenotype is highly heterogeneous, and while there are several common characteristics, there is no single universal molecular senescence marker (Hernandez-Segura et al., 2017). This variability can be driven by a number of factors, such as the senescence-triggering stimuli, the cell type, or the passage of time. Here, we will discuss the molecular and structural alterations that occur within senescent cells (Figure 1).

#### 3.2.1 DNA damage

Aging cells are exposed to a plethora of stressors that can cause DNA damage and consequently trigger the DDR. This damage can be in the form of single- or double-stranded breaks (SSB and DSB, respectively) which, if left unresolved, will lead to cellular senescence (Kumari and Jat, 2021). While there are a multitude of different stimuli that can prompt DNA damage, it is usually the p53/p21^CIP1^ pathway that is initiated in response (Fumagalli et al., 2012). Persistent DDR signalling leads to p53 phosphorylation and p21 activation, which in turn inhibits cyclin-dependent kinase 2 (CDK2) (Campisi and D’Adda Di Fagagna, 2007). This causes the hypophosphorylation of retinoblastoma (RB), a tumour suppressor protein, that ultimately leads to cell cycle arrest and senescence (Chicas et al., 2010). Another CDKI, p16^INK4a^, accumulates with age and is generally implicated in replicative senescence signalling. Like p21, p16 also hypophosphorylates RB to halt cell proliferation. Senescent growth arrest was traditionally thought to occur in the G1 phase of the cell cycle, but several studies have now shown that G2 exit can occur with p21-mediated DNA damage signalling (Gire and Dulić, 2015).

#### 3.2.2 Apoptotic resistance

A key cellular mechanism present in many senescent cells is apoptotic resistance. Apoptosis is the programmed and highly regulated process of cell death. Like senescence, apoptosis is routinely initiated in response to extreme cellular stress. The mechanism that dictates which of these processes a damaged cell will experience is poorly understood, but one hypothesis is that acute DNA damage initiates apoptosis while sustained DNA damage induces senescence (Kumari and Jat, 2021). Notably, senescent cells often exhibit robust anti-apoptotic mechanisms to prolong their stability and lifespan, which could explain why these cells accumulate with age (Campisi and D’Adda Di Fagagna, 2007). A class of genes known as senescence-associated super-enhancers (SASE) have recently been linked to promoting the survival of senescent cells (Sturmlechner et al., 2022). In response to DNA damage, the SASE members *Mdm2, Rnas4,* and *Ang* act to suppress p53-mediated apoptosis and preserve cellular senescence (Sturmlechner et al., 2022). At first glance, apoptosis and senescence may seem like opposing cell fates; however, these processes work together to contribute to the dysfunction of aging tissues. In fact, the primary class of therapeutics aimed at combating cellular senescence–senolytics–target the anti-apoptotic pathways of senescent cells (see Section 5).

#### 3.2.3 Metabolic alterations

The development of apoptosis resistance is accompanied by alterations in senescent cell metabolic activity. Common metabolic alterations include defects in OXPHOS and a transition to glycolytic energy systems. Senescence triggers the significant metabolic reprogramming of a cell, but these changes are highly heterogeneous and will vary based on the cell type and mode of senescence induction. For example, human fibroblasts induced to senescence via irradiation or replication have elevated glycolysis and reduced oxidative activity, while oncogene-induced senescent fibroblasts activate both glycolysis and OXPHOS (Takebayashi et al., 2015; James et al., 2015). Oddly, increased glycolysis has been shown to protect cells from premature senescence (Kondoh et al., 2005). While the metabolic activity of senescent cells is certainly modified, the large variability in physiological response makes it challenging to use metabolic parameters as universal senescence biomarkers. Importantly, senescent cells are also capable of metabolically reprogramming adjacent, non-senescent cells through the actions of the SASP (discussed further below) (Acosta et al., 2013).

#### 3.2.4 Loss of proteostasis

Moreover, senescent cells experience a loss of proteostasis, primarily characterized by dysfunctions in autophagy. Senescent cells tend to accumulate dysfunctional lysosomes over time, a feature that is the basis for the senescence-associated β-galactosidase (SA-β-gal) assay (Dimri et al., 1995). These organelles are key components of autophagy as they regulate the degradation and recycling of unneeded cellular material (Yim and Mizushima, 2020). Furthermore, senescence often results in the elevated activity of mechanistic target of rapamycin complex 1 (mTORC1), which suppresses autophagy and restricts the cell’s ability to clear damaged components (Kang et al., 2011; Tai et al., 2017). Mitophagy is also presumably suppressed by the over-activation of mTORC1, which would reduce the clearance of dysfunctional mitochondria and exacerbate the proteostatic aberrations observed in senescent cells (Korolchuk et al., 2017).

#### 3.2.5 Morphological changes

Cellular senescence often presents with several morphological changes that can be easily detected *in vitro.* Senescent cells generally exhibit an enlarged and abnormally shaped cell body, potentially due to the formation of actin stress fibres (Huang et al., 2022). Furthermore, senescent signalling can activate sodium channels on the plasma membranes of cells, elevating intracellular sodium concentrations (Warnier et al., 2018). This, in conjunction with the senescence-mediated upregulation of caveolin-1, ultimately results in plasma membrane destabilization (Cho et al., 2004; Chrétien et al., 2008; Dasari et al., 2006). More internally, senescent cells often have destabilized nuclei due to reduced Lamin B1 protein on the inner nuclear membrane, which causes chromatin remodelling and results in enlarged nuclei or multinucleation (Freund et al., 2012; Shimi et al., 2011). While these characteristics are readily identifiable *in vitro,* senescent cells *in vivo* usually retain a normal morphology dictated by their surrounding environment (Muñoz-Espín and Serrano, 2014). Mitochondrial structure is also severely impacted by cellular senescence. Typically, senescent cells have larger and more abundant mitochondria than their healthy counterparts due to blunted mitophagy that allows for the accumulation of older, dysfunctional organelles (Tai et al., 2017; Korolchuk et al., 2017; Hara et al., 2013). These mitochondria experience diminished membrane potential and have increased ROS production, leading to oxidative stress and further exacerbating the senescent phenotype (Passos et al., 2007).

### 3.3 The senescence-associated secretory phenotype (SASP)

Cellular senescence is not only detrimental to the affected cell, but also to the cellular microenvironment due to the actions of the SASP. This feature is capable of intercellular communication and signalling through the components released by the senescent cells. Traditionally, the SASP was characterized by the secretion of several protein types, such as cytokines, chemokines, growth factors and proteases (Coppé et al., 2008), but over the years, other molecules have been added to this list, including bioactive lipids, extracellular vesicles, and non-coding nucleic acids (Table 1) (Wang et al., 2024). While all of these components have been identified in the secretome of senescence cells, the exact composition of the SASP will vary based on cell type and the intracellular senescent phenotype present at that time (Coppé et al., 2010; Wiley et al., 2017; Basisty et al., 2020).

**TABLE 1:** Senescence-associated secretory phenotype (SASP) components.

Category	Class	SASP components
Proteins	Interleukins/cytokines	IL-1α, IL-1β, IL-6, IL-7, IL-8, IL-11, IL-13, IL-15, IL-33, BAFF
	Chemokines	CCL2, CCL3, CCL5, CCL8, CXCL1, CXCL2, CXCL3, CXCL5, CXCL8, CXCL10, CXCL11, CXCL14, GRO-a, GRO-b, GRO-g, MCP-2, MCP-4, MIP-1a, MIP-3a, HCC-4, eotaxin, eotaxin-3, TECK, ENA-78, I-309, I-TAC
	Growth factors	TGFβ, GDF15, amphiregulin, epiregulin, heregulin, EGF, bFGF, HGF, KGF (FGF7), VEGF, angiogenin, SCF, SDF-1, PIGF, NGF, IGFBP2, IGFBP3, IGFBP4, IGFBP6, IGFBP7
	Proteases and regulators	MMP1, MMP3, MMP10, MMP12, MMP13, MMP14, TIMP1, TIMP2, PAI-1, PAI-2, tPA, uPA, cathepsin B
	Insoluble proteins	Fibronectin, collagen, laminin
	Others	eNOS, LIF, ISG15
Non-protein molecules		ROS, NO, PGE2
Lipids		Prostaglandins, leukotrienes
Other structures		Extracellular vesicles, miRNAs, cytoplasmic chromatin fragments

*Table adapted from* (Wang et al., 2024; Gorgoulis et al., 2019).

#### 3.3.1 Protein-based factors

The most prominent and well-characterized component of the SASP is the proteins, which can be classified into three categories–soluble signalling proteins, proteases, and insoluble proteins. Soluble signalling proteins are a large source of bioactive molecules that have the potential to influence several signal transduction pathways related to tumour progression, inflammation, and other age-related diseases (Matsuda et al., 2023; Zhou et al., 2022; Ortiz-Montero et al., 2017). Notable members of this category include interleukins, chemokines, and growth factors (Wang et al., 2024; Matsuda et al., 2023; Orjalo et al., 2009; Severino et al., 2013). Proteases, which are responsible for the degradation of signalling proteins and the extracellular matrix (ECM), are also released as SASP factors (Liu and Hornsby, 2007). Lastly, insoluble proteins, such as the cell surface protein fibronectin, are often released by senescent cells, resulting in significant cytoskeletal remodelling (Chen et al., 2006).

#### 3.3.2 Lipids and extracellular vesicles

Additional SASP components with prominent bioactive properties have also been identified. Certain lipids, such as phospholipids or oxidized fatty acids, are released by senescent cells and can promote fibrosis, inflammation, and cell cycle arrest (Narzt et al., 2021; Wiley et al., 2019; Wiley et al., 2021). Additionally, small extracellular vesicles (EVs) released by senescent cells have been shown to affect cell proliferation and apoptotic signalling (Takasugi et al., 2017; Terlecki-Zaniewicz et al., 2018). EVs contain proteins, lipids, and non-coding nucleic acids such as microRNAs (miRNA) and can regulate intercellular senescence communication (Takasugi, 2018). MiRNAs, which post-transcriptionally regulate gene expression, have often been identified as the primary drivers of EV-mediated senescence signalling (Disayabutr et al., 2016; Lee et al., 2023; Fabian et al., 2010; Valadi et al., 2007). Finally, there are several SASP components that are not classified under any of these categories but still have significant roles in regulating senescence-associated signalling. These include nitric oxide (NO) and ROS, which are produced because of the altered metabolism and oxidative stress present in senescent cells (Wang et al., 2024; Mabrouk et al., 2020).

#### 3.3.3 Regulation of the SASP

The SASP is driven by multiple intracellular senescence signalling pathways, primarily those involved in the DDR (Wiley and Campisi, 2021; Rodier et al., 2009). While the current understanding of SASP regulation is limited, several mediators of the phenotype have been identified, including p53, p16, NF-κB, C/EBPβ, p38 MAPK, mTOR, and NOTCH (Coppé et al., 2008; Freund et al., 2011; Herranz et al., 2015; Acosta et al., 2008; Hoare et al., 2016; Buj et al., 2021). The SASP of DNA-damaged cells is primarily controlled by the transcription factors NF-κB and C/EBPβ, which regulate the release of interleukins, chemokines, and growth factors (Acosta et al., 2008; Chien et al., 2011). However, other key signalling molecules have also been implicated in SASP regulation. The depletion of p53 has been shown to increase the release of pro-inflammatory factors, while p16 suppression decreases the expression of multiple SASP components (Buj et al., 2021; Wiley et al., 2016). Moreover, p38 MAPK can trigger a SASP profile by activating NF-κB independent of the DDR, while mTOR promotes SASP expression by differentially regulating the translation of MK2 through 4EBP1 (Freund et al., 2011; Herranz et al., 2015). Finally, NOTCH1 activation drives a TGFβ-mediated secretome while inhibiting the C/EBPβ-mediated SASP (Hoare et al., 2016).

Short-term exposure to SASP factors can have beneficial effects, such as in wound healing or during embryonic development, but the presence of a chronic SASP can have major implications, especially for aging tissues (Demaria et al., 2014; Muñoz-Espín et al., 2013). Chronic SASP activity can have detrimental effects by causing the remodelling of the ECM, increasing fibrosis, or propagating apoptosis in otherwise healthy tissues (Wang et al., 2024). The SASP is not only a consequence of cellular senescence, but it also acts as a senescence inducer; secreted factors can propagate paracrine senescence on neighbouring, healthy cells (Faget et al., 2019). Furthermore, the SASP causes the rise of chemokine and cytokine levels in the cellular microenvironment, contributing to the chronic state of inflammation present in advanced age (Li et al., 2023). The targeted removal of senescent cells and the accompanying SASP improved physical function and extended the lifespan of aged mice, suggesting a promising therapeutic approach to combat aging-related dysfunction (Xu et al., 2018).

## 4 The pathophysiology of senescence within aged muscle

**FIGURE 2:**
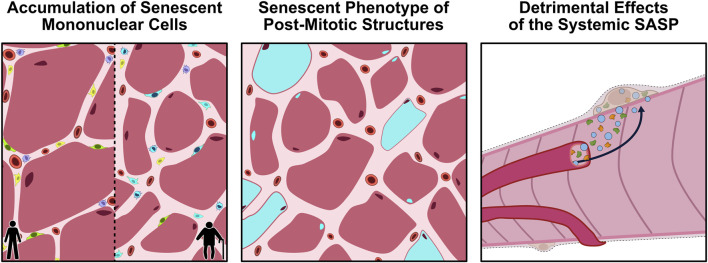
Proposed avenues through which cellular senescence could influence aging skeletal muscle. *Panel 1*: With aging, senescent mononuclear cells (blue) accumulate within the muscle microenvironment, leading to a loss of normal cellular function and impairments in muscle regeneration. *Panel 2:* Emerging evidence suggests that even post-mitotic structures, such as myofibers and myonuclei, may acquire a senescence-like phenotype (blue), characterized by persistent DNA-damage signalling, mitochondrial dysfunction, and secretion of SASP factors. *Panel 3*: The chronic SASP produced by tissue-resident and circulating senescent cells can enter the systemic circulation, propagating inflammation and dysfunction in other tissues, such as skeletal muscle. Together, these processes can compromise the homeostatic status of aging skeletal muscle. SASP, senescence-associated secretory phenotype.

In recent years, there has been a growing interest in the potential for cellular senescence to be implicated in the aging-related perturbations of skeletal muscle. Early work by Baker et al. (2008) found that the systemic inactivation of p16 in BubR1-insufficient mice attenuated the degenerative loss of muscle mass and strength observed in these animals. Since then, there have been multiple investigations directly and indirectly examining the impact of cellular senescence on muscle physiology. There are three possible avenues through which senescence could elicit its effects on aged skeletal muscle: 1) the accumulation of senescent mononuclear cells in skeletal muscle; 2) the induced senescent phenotype of post-mitotic muscle components (*i.e.*, myofibres and myonuclei); and 3) the detrimental effects of the SASP caused by senescent cells in other tissues (Figure 2). This section will discuss the available literature related to each of these theories and the potential that they are implicated in the aging-related dysfunction of skeletal muscle.

### 4.1 Accumulation of senescent mononuclear cells

#### 4.1.1 Evidence supporting senescent cell accumulation

Senescent cells are known to accumulate in multiple tissues with aging, which is generally believed to be detrimental to the overall health and function of our bodies (Baker et al., 2008). Several studies have found evidence that senescent cell markers increase with aging in both rodent and human skeletal muscles (Zhang X. et al., 2022; Hudgins et al., 2018; Sosa et al., 2018; Da Silva et al., 2019). While most of this work examined cellular senescence in whole muscle, others have directly examined the muscle-resident mononuclear cell populations, such as in satellite cells (MuSC), the predominant muscle stem cell responsible for repair and regeneration. In aged muscle, multiple studies have shown that MuSCs express elevated levels of SA-β-gal, p16, and p21 (García-Prat et al., 2016b; Li et al., 2015; Sousa-Victor et al., 2014; Cosgrove et al., 2014; Zhu et al., 2019). While these observations suggest that MuSCs may be accumulating in skeletal muscle over time, one study did show that senescence is apparent only in extremely old (geriatric) MuSCs but not in young or old muscles, indicating that these cells might be accumulating genomic damage over time that only presents as senescence in very advanced ages (Sousa-Victor et al., 2014). Early work investigating muscle cell senescence also observed an accumulation of p16+ fibro-adipogenic progenitors (FAPs) in BubR1 progeroid mice (Baker et al., 2013). More recently, FAPs in aged muscle have been observed to have elevated expression of p16 (Zhang X. et al., 2022). Notably, this p16-positive FAP population had a transcriptional profile highly consistent with senescent gene signalling, providing compelling evidence that these cells were likely senescent (Zhang X. et al., 2022). Additionally, CD68^+^ macrophages and mesenchymal progenitor cells in the interstitium of skeletal muscle have elevated levels of p16 and γH2AX, respectively (Sugihara et al., 2018; Guzman et al., 2022). Others have detected elevated senescence markers in aged muscle cells, but these authors did not quantify the cell type, so it is not possible to know which populations are accumulating with age (Da Silva et al., 2019; Baker et al., 2016).

Several potential explanations exist for why muscle cells are experiencing elevated levels of senescence over time. Nearly 60% of MuSCs isolated from aged muscle or identified on isolated aged myofibres showed increased expression of γH2AX relative to their young counterparts, indicating that these cells are routinely exposed to DNA damage (Sinha et al., 2014). Furthermore, aged MuSCs exhibit a significant impairment of autophagy which reduces the clearance of dysfunctional organelles and causes the build-up of toxic cellular waste (García-Prat et al., 2016b). This results in the accumulation of damaged mitochondria that are constantly producing substantial amounts of ROS and thus promoting oxidative stress (Hong et al., 2022). Old MuSCs have also demonstrated a failure to suppress p16 activity and consequently experience an impaired myogenic capacity followed by senescence induction, which has been linked to the activation of p38 MAPK and the reduction of H2AK119Ub-mediated silencing (Sousa-Victor et al., 2014; Cosgrove et al., 2014). Other potential causes include metabolic disruptions, such as hyperphosphatemia, which can promote senescence in myoblasts by impairing autophagic mechanisms, and miRNAs, which have been implicated in aging muscle and shown to alter senescent gene expression (Sosa et al., 2018; Soriano‐Arroquia et al., 2021; Hu et al., 2014).

#### 4.1.2 Evidence against senescent cell accumulation

Unfortunately, the literature contains many conflicting reports regarding the accumulation of senescent cells in aging skeletal muscle. Several studies by the Peterson laboratory have found no difference in senescence markers between young and old skeletal muscle in both mice and humans (Dungan et al., 2021; Dungan et al., 2020; Dungan et al., 2022). These observations were confirmed by other groups who detected a very low abundance of SA-β-gal–positive cells in old muscle (Moiseeva et al., 2023; Lai et al., 2024). Furthermore, human muscle precursor cells isolated from young and old donors have been found to have negligible differences in their senescent profile (Alsharidah et al., 2013); some research has even shown that MuSCs have no change in p16 expression with aging (Zhang X. et al., 2022; Baker et al., 2013). Taken together, these observations suggest that senescent cells do not accumulate in aging skeletal muscle, in contrast to the work discussed earlier.

There are some potential explanations for the discrepancy in senescent cell accumulation within aged muscle. One theory is that the expression of these common senescence biomarkers is incredibly low. Skeletal muscle inherently has very low quantifiable γH2AX, p16, and p21 expression, while SA-β-gal staining was very rare in both young and old resting muscles (Dungan et al., 2020; Dungan et al., 2022; Moiseeva et al., 2023; Lai et al., 2024; Wang et al., 2009). The low expression of senescent biomarkers can make it challenging to accurately quantify cellular senescence and lead to differential results between studies, especially since a significant proportion of the literature utilizes a single marker, which is not the preferred approach (Gorgoulis et al., 2019). When multiple senescence markers are used instead, several studies have identified an accumulation of senescent cells in aging skeletal muscle (Zhang et al., 2022a; Da Silva et al., 2019). However, this is not always the case, as the combined use of SA-β-gal and p21 failed to detect an elevation in aged mouse muscle (Dungan et al., 2022). It is possible that this observation was affected by the fact that p21 is highly upregulated during MuSC myogenesis and thus may not be ideal for quantifying senescence in skeletal muscle (Hawke et al., 2003). Another potential explanation for the conflicting observations in aged skeletal muscle is related to the definition of “old age”. In one study, “old” mice aged 20–24 months had no change in p16+ MuSCs, while “geriatric” mice aged 28–32 months showed an increase (Sousa-Victor et al., 2014). This could also explain why some human research in adults aged 75 ± 3 found no change in senescence accumulation compared to young individuals, while other studies looking at older adults aged 83 ± 7 observed a change (García-Prat et al., 2016b; Dungan et al., 2020). Lastly, a recent review suggested that other factors, such as animal facility location, sex, fibre type composition, or spatial location of the senescent cells might also account for these discrepancies in aged skeletal muscle (Dungan et al., 2023). Emerging technologies, such as spatial transcriptomics, may soon help overcome some of the challenges with senescence characterization (such as multi-marker labelling) and reveal a more definitive picture of senescent cell accumulation with aging.

### 4.2 Senescent features within post-mitotic structures

Traditionally, senescence is thought to occur exclusively in mitotically capable cells, but there is a growing body of evidence suggesting that post-mitotic senescence could be possible (Sapieha and Mallette, 2018; Von Zglinicki et al., 2021). Several terminally differentiated cells have been found to accumulate multiple senescence markers with aging, including neurons, cardiomyocytes, cochlear cells and osteocytes (Jurk et al., 2012; Anderson et al., 2019; Farr et al., 2016; Benkafadar et al., 2019). The vast majority of skeletal muscle is composed of terminally differentiated myofibres and post-mitotic myonuclei, so the implication of senescence in these structures is quite significant. Human RNA-seq data from the Genotype-Tissue Expression (GTEx) project showed that p16 expression is significantly elevated in multiple tissues, including skeletal muscle (Hudgins et al., 2018). This has since been confirmed directly through RT-PCR, with old male individuals exhibiting 2- to 5-fold greater p21 and p16 mRNA expression in skeletal muscle biopsies relative to young males (Balan et al., 2020). Meanwhile, numerous studies have found that aged mice have elevated levels of p53, p21, and p16 in myofibres compared to young mice (Zhang X. et al., 2022; Sosa et al., 2018; da Silva et al., 2019; Solovyeva et al., 2021). These myofibres also have increased expression of pro-inflammatory SASP genes, including IL-1α, Il-1β, IL-6, and TNF-α (Da Silva et al., 2019). Alternatively, conflicting reports show decreased p21 protein in aged human skeletal muscle and the absence of elevated p16 mRNA in mature myofibres from BubR1 progeroid mice (Baker et al., 2013; Dethlefsen et al., 2018). Taken together, these observations indicate the potential that aged myofibres and/or their myonuclei could experience a molecular signature indicative of senescence.

The notion that post-mitotic myonuclei might be senescent is exciting. Previous work has identified senescent nuclei in aged skeletal muscle, but unfortunately, many of these studies did not utilize additional biomarkers to determine the source of these cells (Dungan et al., 2023). Myonuclei are capable of accumulating DNA damage when metabolically stressed, such as during obesity (Dungan et al., 2020). Expanding on this leads us to theorize that myonuclei could undergo mitotically-independent senescence by activating the DDR. Notably, two independent single-nuclei RNA-seq-based studies identified subpopulations of myonuclei that have elevated p21 gene expression exclusively in aged muscle samples (Zhang X. et al., 2022; Perez et al., 2022). Moreover, the myofibres of old mice contain more p21-positive myonuclei with higher levels of telomere-associated DNA damage foci (TAF), suggesting the presence of senescence in these nuclei (Da Silva et al., 2019). These observations are nicely complimented by recent evidence showing that mouse myonuclei are capable of synthesizing DNA and endoreplication (Borowik et al., 2022). These results indicate that at least a portion of myonuclei might not be post-mitotic and could experience a senescence-like phenotype. The induction of senescence within aged myofibres and/or myonuclei could have substantial effects on skeletal muscle homeostasis.

### 4.3 Systemic SASP cross-tissue effects on skeletal muscle

Lastly, there is a strong potential for systemic SASP factors to contribute to the altered physiology of aged skeletal muscle. The SASP, as discussed above, is an impactful characteristic of senescent cells that can exert an effect on both the cellular microenvironment and distant tissues (Coppé et al., 2010). In acute exposure, SASP factors are essential for homeostatic processes like wound healing and tumour suppression, however, chronic SASP production is associated with several detriments to tissue health (Coppé et al., 2010; Birch and Gil, 2020). The SASP factor CCL17 contributes to multi-organ fibrosis in the heart, aorta, and vascular endothelium (Zhang et al., 2023; Zhang Y. et al., 2022). Additionally, hepatocellular senescence can induce multi-tissue senescence and dysfunction through TGF-β signalling pathways, which can be reversed with senolytics that selectively eliminate senescent hepatocytes, highlighting the potential for systemic SASP effects originating from a single senescent cell type (Du et al., 2025; Kiourtis et al., 2024). During aging, we typically observe numerous adverse changes within skeletal muscle, including excessive inflammation, ECM remodelling, increased fibrosis, and altered intercellular communication–all of which are commonly associated with chronic SASP exposure (Englund et al., 2021; He et al., 2021). While it seems reasonable that the SASP of other tissues is, at least partially, contributing to these perturbations, this is an area of research that needs substantially more investigation.

The SASP signature is a moving target–various factors, such as cell type and means of senescence induction, can influence the factors that are secreted. Its composition has even been noted to change over a cell’s progression through senescence (Coppé et al., 2010; Wiley et al., 2017; Basisty et al., 2020). This makes it difficult to identify key SASP molecules or proteins that elicit their effects on skeletal muscle health and homeostasis during aging. However, certain SASP components appear to be consistent across tissues and species with high fidelity, and importantly, several of these factors are known to mediate skeletal muscle adaptations (Saul et al., 2022).

#### 4.3.1 Pro-inflammatory SASP factors

Perhaps the most prominent and well-characterized aspect of the SASP is the pro-inflammatory factors. Given that inflammation is considered a key hallmark of aging muscle, it follows that the SASP could be a potential cause of the observed chronic inflammatory state (Coppé et al., 2010; Franceschi et al., 2018). Several studies have identified elevated levels of inflammation-related SASP factors within aged muscle (Zhang et al., 2022a; Da Silva et al., 2019; Moiseeva et al., 2023). In line with this, elevated mRNA expression of inflammatory proteins IL-1α, IL-1β, IL-6, and TNF-α have been detected within the skeletal muscle of geriatric mice (32-month-old), which was accompanied by decreases in fibre size, increases in centrally located nuclei, and greater expression of senescence markers (da Silva et al., 2019). It is also likely that circulating pro-inflammatory SASP factors originating from other tissues contribute to the chronic inflammatory state of aged muscle. Some of the strongest evidence for this comes from senescent cell transplantation experiments. Xu et al. (2018) used luciferase-expressing senescent preadipocytes with a SASP profile resembling endogenously aged senescent cells, such as the cytokines IL-6 and TNF-α, and injected them intraperitoneally into young mice (Xu et al., 2018). Two months later, luciferase-negative senescent cells and biomarkers were observed in multiple tissues, including the quadriceps muscles, indicating the recipient’s own cells underwent cellular senescence in response to SASP signals from the transplanted adipocytes (Xu et al., 2018). SASP components IL-6 and TNF-α are of particular interest as their chronically elevated expression with age is associated with several phenotypes, such as greater muscle wasting and protein catabolism, and reductions in protein synthesis rates, muscle mass and strength (Reid and Li, 2001; Schaap et al., 2006; Visser et al., 2002). This presents a plausible role for key pro-inflammatory SASP molecules like IL-6 and TNF-α in the functional decline of muscle in aging.

On a cellular level, inflammatory SASP factors have been shown to significantly impact MuSC dynamics. The transplantation of induced senescent cells into skeletal muscle resulted in DNA damage in endogenous MuSCs and increased immune cell recruitment, fibrosis, and delayed regeneration (Moiseeva et al., 2023). Moreover, TNF-α is known to impair myoblast differentiation in culture, disrupt MuSC regenerative capacity, and influence muscle atrophy through NF-κB-mediated signalling (Reid and Li, 2001; Guttridge et al., 2000; Layne and Farmer, 1999). It is also possible that inflammatory SASP components can interact with the MuSC niche, potentially dysregulating stem cell activity. Factors such as TGF-β, which promote ECM deposition, could thicken the basal lamina and impair MuSC communicative abilities (Gopinath and Rando, 2008; Ismaeel et al., 2019). Importantly, the myocytes and MuSCs of aged mice have elevated TGF-β levels that are associated with the blunted regenerative capacity of aged MuSCs (Carlson et al., 2008). Thus, there is evidence to suggest that SASP inflammatory components can interact with MuSCs through paracrine signalling, potentially disrupting them enough to contribute to their observed dysfunction with age.

#### 4.3.2 Senescence-associated metabolites

An emerging area of research has identified that there are metabolites, such as bioactive lipids and amino acids, that are important proponents of the SASP. Preliminary investigations into the metabolic signature of senescence suggest a highly dynamic and heterogeneous metabolome occurs with senescence induction (Kamal et al., 2025; Lopes-Paciencia et al., 2019; Pundlik et al., 2024). This seems reasonable, given that a major process like cell cycle arrest is likely to influence cellular metabolism. Indeed, oncogene-activated senescent fibroblasts have elevated β-oxidation and fatty-acid breakdown (Quijano et al., 2012). Certain metabolites have been shown to modulate recipient cells in a SASP-like manner, but it is evident that others do not elicit any paracrine effects on the cellular microenvironment (Kamal et al., 2025; Lopes-Paciencia et al., 2019; Pundlik et al., 2024). Among the bioactive metabolites, lipids have garnered the most evidence as key SASP members within skeletal muscle. Some fatty acids, like arachidonic acid and oleic acid, are found at elevated concentrations within senescent cells and in their extracellular environment (Kamal et al., 2025; Lopes-Paciencia et al., 2019; Pundlik et al., 2024; Harizi et al., 2008). Inhibition of arachidonic acid-derived 15d-PGJ2 from senescent myoblasts restores muscle differentiation, highlighting the potential for bioactive lipids and their products to regulate muscle homeostasis (Pundlik et al., 2024). While these data suggest that lipids may directly influence muscle properties, it is possible that they act by stimulating the production of inflammatory SASP factors. Treating healthy myoblasts with oleic acid upregulates prominent SASP genes CCL2, CXCL12, and IL-33 (Kamal et al., 2025). Moreover, lipid depletion within senescent fibroblasts drastically reduces the production of the SASP components IL-6, CXCL-1, CXCL-10, and CXCL-5 (Ogrodnik et al., 2019). While it is important to note that most of these findings were conducted using *in vitro* models, they still highlight the potential for metabolites to play a key role in the expression of the SASP within aging skeletal muscle.

#### 4.3.3 Extracellular vesicles

Another SASP component emerging as a potential mediator of muscle physiology during aging are extracellular vesicles (EVs). Serum extracted from young mice and introduced to old mice has been shown to improve muscle regeneration, but these effects were lost when the blood was depleted of EVs (Sahu et al., 2021). This has also been observed in mouse and human fibroblasts, where EVs isolated from young fibroblasts ameliorated senescence in old ones by increasing their antioxidant capacity (Fafián-Labora et al., 2020). EVs have been linked to SASP regulation, primarily through their miRNA cargo, and can affect cellular processes throughout the body via autocrine and paracrine signalling (Anakor et al., 2021; Tanaka and Takahashi, 2021). They are also thought to promote immune senescence during aging through self-contained inflammatory miRNAs (Olivieri et al., 2015). These small structures are released in large quantities following DNA damage and senescence induction and have the potential to affect multiple tissues during aging, including skeletal muscle (Tanaka and Takahashi, 2021; Lehmann et al., 2008; Alfonzo et al., 2022).

In summary, the SASP likely plays a role in the dysfunction of muscle physiology during advanced age. While pro-inflammatory SASP factors, bioactive metabolites, and EVs are promising candidates for eliciting a senescent response in aged skeletal muscle, many details remain unclear. For instance, the extent to which systemic SASP factors influence muscle homeostasis is not known; the majority of these effects may originate from neighbouring muscle cells rather than other tissues. Additional studies are needed to answer these questions, and investigations into anti-SASP therapies (i.e., senomorphics) could help us better understand the implications this feature has on aging muscle.

## 5 Senotherapeutic strategies to counteract muscle aging

Since the accumulation of senescent cells is a hallmark of aging, our ability to target and eliminate them is crucial. The transgenic removal of senescent cells from various tissues, including the eyes, skeletal muscle, and adipose, delayed the onset of various aging-related pathologies and extended the lifespan in mice (Baker et al., 2011). Furthermore, aged p16^INK4a^–null mice had larger muscle fibres and improved exercise performance relative to their non-transgenic counterparts, suggesting that the elimination of senescent cells may be a viable approach to counteract aging-related muscle perturbations (Baker et al., 2011). First introduced in 2015, senotherapeutics represent a new class of drugs that aim to reduce the impact of cellular senescence and can be classified as either senolytics or senomorphics (Zhu et al., 2015). The key difference between these two classes is that senolytics directly target and eliminate senescent cells while senomorphics aim to alleviate the effects of the SASP (Zhu et al., 2015). Senolytics primarily act by transiently disabling the senescence cell anti-apoptotic pathway (SCAP) to promote the selective elimination of senescent cells (Kirkland and Tchkonia, 2020). Alternatively, the mechanisms governing senomorphics can vary significantly based on the targeted SASP component and are not as well-studied as senolytics in the context of skeletal muscle aging.

**FIGURE 3:**
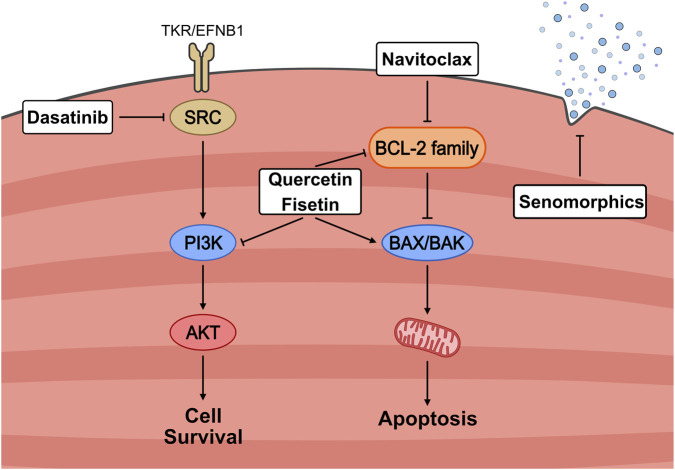
Mechanistic overview of senotherapeutic strategies targeting senescent cell survival pathways. Senescent cells resist apoptosis through the activation of pro-survival signalling cascades, including SRC/PI3K/AKT and BCL-2 family members. Senolytic agents selectively disrupt these defenses: *Dasatinib* inhibits SRC signalling, *Quercetin* and *Fisetin* suppress PI3K and BCL-2 pathways, and *Navitoclax* directly antagonizes anti-apoptotic BCL-2 proteins, thereby promoting mitochondrial-mediated apoptosis via BAX/BAK activation. In contrast, senomorphics act to attenuate the senescence-associated secretory phenotype (SASP) without inducing cell death. Together, these approaches aim to reduce the burden or harmful effects of senescent cells in aging muscle.

The first major development in senolytics was the discovery that dasatinib and quercetin (D + Q) could target pro-survival pathways and promote apoptosis in senescent cells (Zhu et al., 2015). These compounds were identified through bioinformatic analyses to determine clinically approved products that could target the SCAP network (Kirkland and Tchkonia, 2020). Dasatinib was an FDA-approved chemotherapeutic that inhibited tyrosine kinase, and quercetin was a naturally occurring flavonoid found in apple peels. The combination of D + Q has been repeatedly shown to be effective at inducing apoptosis in all types of senescent cells and is arguably the most widely used senolytic (Dungan et al., 2022; Zhu et al., 2015; Alharbi et al., 2022). Soon after, other promising senolytics were identified, such as ABT263, more commonly known as Navitoclax, which acts on the pro-survival BCL2 protein family that is naturally elevated within senescent cells to protect against apoptosis (Zhu et al., 2016; Wang, 1995; Chang et al., 2016). Natural senolytic compounds like the flavonoids quercetin and fisetin inhibit the BCL2 family and certain SCAP members (Zhu et al., 2017; Yousefzadeh et al., 2018). There are currently dozens of validated and predicted senolytic compounds, but this review will focus on a handful that have been adequately investigated within skeletal muscle, namely, Navitoclax, fisetin, dasatinib and quercetin (Figure 3).

### 5.1 *In vitro* potential of senolytic drugs

Senolytic compounds are often first examined through *in vitro* models of senescence. Navitoclax has been shown to selectively kill senescent cells independent of cell type, senescence induction method, or species, except for human primary pre-adipocytes (Zhu et al., 2016; Chang et al., 2016). Within the context of skeletal muscle, Navitoclax can effectively clear senescent muscle progenitor cells (MPCs) isolated from naturally aged mice and improve the number of non-senescent MPCs *in vitro* (Chang et al., 2016). While these results are promising, there is limited evidence for Navitoclax’s effectiveness at eliminating senescent muscle cells *in vivo*. The flavonoid fisetin has also been shown to be an effective senolytic for muscle-derived cells from Z24^−/−^ progeroid mice (Liu et al., 2022a). Senescent FAPs isolated from these mice impair the proliferation and myogenic potential of co-cultured wildtype MPCs, but these effects can be reversed with acute fisetin treatment. In C_2_C_12_ mouse myoblasts, fisetin significantly attenuated H_2_O_2_-induced oxidative stress and DNA damage while limiting ROS generation, all of which are known senescence inducers (Park et al., 2023). Moreover, multiple studies have reported that fisetin can alleviate senescence within vascular smooth muscle cells through the PTEN-mTORC2 signalling axis (Kim et al., 2021; Kim et al., 2023a; Kim et al., 2023b). Similarly, quercetin, either independently or in combination with dasatinib, can induce apoptosis in senescent vascular smooth muscle cells (Gadecka et al., 2025; K et al., 2020). Overall, there is ample evidence that senolytic agents are highly effective at clearing senescent muscle cells in culture, and they offer reliable experimental models for investigating the underlying mechanisms of cellular senescence in muscle.

### 5.2 Senolytics during muscle regeneration

Aged skeletal muscle has a reduced capacity for repair and regeneration, and treatments that improve these processes could extend healthspan and quality of life in the elderly. Senolytics can have varying effects in the skeletal muscle microenvironment, especially during periods of muscle damage. There is evidence that muscle-resident cells involved in muscle repair, namely, MuSCs, FAPs, and macrophages, can be influenced by senolytic interventions. Four weeks of treatment with fisetin in adult Z24^−/−^ progeroid mice increased the number of MuSCs while reducing the number of FAPs and intramuscular fibrosis and ultimately improving muscle size and strength (Liu et al., 2022a). Conversely, D + Q treatment in aged mice did not affect MuSC content or intramuscular fibrosis following BaCl_2_ injury (Dungan et al., 2021). In line with this, old mice provided with D + Q for 4 months displayed fewer centrally nucleated myofibres, indicating either an impairment in the MuSC response or a reduced need for muscle regeneration (Zhang X. et al., 2022; Bi et al., 2024). This discrepancy in senolytic impact on MuSCs could be due to the diversity in compound pharmacology, animal models, and injury modalities. Alternatively, the improved muscle phenotype may be driven by the influence of senolytics on other muscle-resident cell types. Seminal work by Zhang et al. found that the administration of D + Q did not attenuate age-related declines in muscle mass or myofibre CSA, but did reduce the number of centrally located fibres and improve grip strength (Zhang X. et al., 2022). However, they reported that the senescent marker p16 was only detectable in FAPs and macrophages, suggesting that these cell types, rather than MuSCs, may be more implicated in the muscle response to senolytics. In the *mdx* model of DMD, short-term treatment with fisetin eliminated senescent macrophages, which in turn improved MuSC function, suggesting that senolytic use can influence muscle regenerative capacity by acting on senescent immune cells (Liu et al., 2022b). Future senotherapeutic research would benefit greatly from evaluating multiple cell populations during various states of muscle damage.

### 5.3 Senolytic impact on aged muscle function

As research supporting a role for senescence in the development of sarcopenia has emerged, so too has an interest in the potential for senolytics to combat skeletal muscle decline with age. Zhu and colleagues (2015) were the first to examine the effects of senolytic use on skeletal muscle. 24-month-old mice were treated with a single dose of 5 mg/kg and 50 mg/kg of D + Q, respectively (Zhu et al., 2015). They found that a single dose of D + Q reduced the number of senescent cells in 24-month-old skeletal muscle, while 6 weeks of senolytic treatment also reduced frailty index and delayed gait disorders in Ercc1^-/Δ^ progeroid mice (Zhu et al., 2015). Later work from this group and others showed that 4 months of D + Q treatment in aged mice (20-month-old) improved several functional metrics, including walking speed, hang time, grip strength, treadmill endurance capacity, motor coordination/balance, and daily activity levels relative to aged vehicle-treated mice (Zhang X. et al., 2022; Xu et al., 2018; Dungan et al., 2021). Another study used SOD1KO mice to model a senescent phenotype by eliciting oxidative stress-induced muscle frailty and found that 7 months of D + Q treatment effectively lowered p21 mRNA expression but did not influence muscle mass, which is in line with previous reports in aged mouse muscle (Zhang X. et al., 2022; Borowik et al., 2024). Despite this, senolytic treatment did increase muscle-specific force generation and improve mitochondrial respiration in these mice (Borowik et al., 2024). Although D + Q is the most common senolytic used in aging muscle research, other drugs such as Navitoclax have also demonstrated potential. Short-term (10 days) Navitoclax treatment in irradiated male C57BL/6 mice resulted in improvements in muscle coordination and endurance capacity (Fielder et al., 2022). Interestingly, these animals had no change in muscle CSA, fibrosis, adiposity, or p21+ cell abundance, indicating that senolytic treatment must have altered muscle function through alternative mechanisms. Overall, senolytics show promise as a potential therapeutic to improve skeletal muscle function with aging, but more research is needed to elucidate their effects and mechanisms of action within *in vivo* models.

## 6 Conclusion

Emerging evidence is linking senescence to several of the perturbations that are present in aged skeletal muscle. Despite some discrepancies in the mechanisms of action, it is clear that this cellular process can regulate aging muscle dynamics, muscle function, and the activity of several muscle-resident cell populations. Recent advances in senotherapeutic research also provide novel strategies for combating or delaying aging. However, our understanding of senescence in the context of skeletal muscle is incomplete, and more work is required to fully elucidate the mechanisms governing senescent regulation of skeletal muscle.
